# Xanthine Oxidase Mediates Hypoxia-Inducible Factor-2α Degradation by Intermittent Hypoxia

**DOI:** 10.1371/journal.pone.0075838

**Published:** 2013-10-04

**Authors:** Jayasri Nanduri, Damodara Reddy Vaddi, Shakil A. Khan, Ning Wang, Vladislav Makerenko, Nanduri R. Prabhakar

**Affiliations:** Institute for Integrative Physiology and Center for Systems Biology of O_2_ Sensing, Biological Sciences Division, University of Chicago, Chicago, Illinois, United States of America; University of Colorado Denver, United States of America

## Abstract

Sleep-disordered breathing with recurrent apnea produces chronic intermittent hypoxia (IH). We previously reported that IH leads to down-regulation of HIF-2α protein via a calpain-dependent signaling pathway resulting in oxidative stress. In the present study, we delineated the signaling pathways associated with calpain-dependent HIF-2α degradation in cell cultures and rats subjected to chronic IH. Reactive oxygen species (ROS) scavengers prevented HIF-2α degradation by IH and ROS mimetic decreased HIF-2α protein levels in rat pheochromocytoma PC12 cell cultures, suggesting that ROS mediate IH-induced HIF-2α degradation. IH activated xanthine oxidase (XO) by increased proteolytic conversion of xanthine dehydrogenase to XO. ROS generated by XO activated calpains, which contributed to HIF-2α degradation by IH. Calpain-induced HIF-2α degradation involves C-terminus but not the N-terminus of the HIF-2α protein. Pharmacological blockade as well as genetic knock down of XO prevented IH induced calpain activation and HIF-2α degradation in PC12 cells. Systemic administration of allopurinol to rats prevented IH-induced hypertension, oxidative stress and XO activation in adrenal medulla. These results demonstrate that ROS generated by XO activation mediates IH-induced HIF-2α degradation via activation of calpains.

## Introduction

Sleep disordered breathing with recurrent apnea (periodic cessation of breathing) produce periodic decreases in arterial blood oxygen levels or chronic intermittent hypoxia (IH). 4–5% of adult males and 2–4% of females after menopause [1] and 50% of infants born preterm [2] are prone to recurrent apnea. Co-morbidities associated with recurrent apnea include hypertension, ventilatory abnormalities, and elevated sympathetic nerve activity [3]. Rodents exposed to chronic IH exhibit autonomic dysfunction similar to that seen in recurrent apnea patients [4]. Recent studies have shown that recurrent apnea patients and chronic IH exposed rodents exhibit elevated levels of reactive oxygen species (ROS) [5]–[8]. Antioxidant treatment prevents chronic IH-induced hypertension and the elevated sympathetic activity in rodents [9] and improves vascular reactivity in recurrent apnea patients [10]. These findings suggest that oxidative stress resulting from elevated ROS levels is a major contributor to the autonomic dysfunction caused by chronic IH.

Recent studies provide important insights into the molecular mechanisms by which chronic IH increases the oxidative stress. Hypoxia-inducible factors (HIFs) are a family of transcriptional activators, which mediate transcriptional responses to decreased availability of oxygen or hypoxia [11]. HIF-1 is the first identified and extensively studied member of the HIF family. HIF-1 is comprised of an O_2_-regulated α subunit and a constitutively expressed β subunit [12]. Chronic IH increases HIF-1α protein levels in rodents and in cell cultures via reactive oxygen species (ROS)-dependent activation of mammalian target of rapamycin (mTOR) and inhibition of HIF-1α hydroxylation [13]. HIF-1 regulates genes encoding pro-oxidant enzymes (NADPH oxidases) [14], [15]. It was proposed that HIF-1 by up regulating the pro-oxidant enzymes contribute in part to the oxidative stress caused by chronic IH [16].

HIF-2, also known as endothelial PAS domain protein-1 (EPAS-1) is another member of the HIF family. HIF-2α subunit shares 48% amino acid sequence identity with HIF-1α [17]. Recent studies showed that HIF-2α expression is differentially regulated by continuous and intermittent hypoxia [18]. While continuous hypoxia increases, IH decreases HIF-2α expression in cell cultures and rodents. The IH-induced HIF-2α degradation is mediated by calpains, especially calpain 1, which is a Ca^2+^-dependent protease [18]. HIF-2α is a potent regulator of the genes encoding anti-oxidant enzymes (AOE) [19]. IH exposed cells exhibit decreased expression of AOEs and oxidative stress. Normalizing HIF-2α levels corrected the AOE expression and prevented IH-induced oxidative stress. Although these findings emphasize an important role for HIF-2α in mediating the IH-evoked oxidative stress, the signaling mechanisms associated with calpain-mediated HIF-2α degradation by IH have not been investigated. In the present study, we delineated the signaling pathways associated with calpain-dependent HIF-2α degradation in cell cultures and in rats subjected to chronic IH.

## Materials and Methods

### Ethics Statement

All animal experiments were approved by the Institutional Animal Care and Use Committee of the University of Chicago (IACUC) (Protocol # 71810).

### Exposure of Cell Cultures to IH

PC12 cells (original clone from Dr. Lloyd Greene, Columbia University Medical Centre, New York, U.S.A) were cultured in Dulbecco’s modified Eagle’s medium (DMEM) supplemented with 10% horse serum, 5% fetal bovine serum (FBS), penicillin (100 U/ml), and streptomycin (100 µg/ml) under 10% CO_2_ and 90% air (20% O_2_) at 37°C [6]. Experiments were performed on cells serum starved for 16 h in antibiotic free medium. In the experiments involving treatment with drugs, cells were pre-incubated for 30 min with either drug or vehicle. Cell cultures were exposed to IH (1.5% O_2_ for 30 sec followed by 20% O_2_ for 5 min at 37°C) as described previously [20]. Ambient O_2_ levels in the IH chamber were monitored by an O_2_ analyzer (Alpha Omega Instruments).

### Exposure of Rats to IH

Adult male rats (Sprague-Dawley, 150–200 gm) were exposed to chronic IH as described previously [21]. Briefly, unrestrained, freely moving rats housed in feeding cages were exposed to 10 days of IH (5% O_2_ for 30 sec followed by 20% O_2_ for 5 min) for 8 h per day. In the experiments involving XO inhibitor, rats were treated with Allopurinol (Calbiochem, 65 mg/kg/day) or vehicle (controls) via oral gavage every day before exposure to daily regimen of IH. Allopurinol suspension was prepared by mixing 2,000 mg allopurinol into a 1% (w/v) methylcellulose suspension to achieve a 20-mg/ml concentration. Placebo was 1%(w/v) methylcellulose suspension.

### Transient Transfections and siRNA Studies

Full length human HIF-2α DNA (pcDNA3.1) was used to generate bHLH and PAS deleted HIF-2α constructs. ΔUR and ΔUR+ΔCTAD HIF-2α constructs were gift from Dr. J.A. Garcia (South Western Medical School, Dallas, TX). Cells were transfected with plasmid DNA using Lipofectamine Plus (Invitrogen) reagent. Transiently transfected cells were starved in serum-free growth medium for 16 h, and then were exposed to IH_._ Following protocol was employed in experiments involving siRNA approach. PC12 cells (5×10^5^) were plated on collagen type IV (BD Biosciences, Bedford, MA) coated culture dishes and cultured for 24 h before transfection with siRNA (Santa Cruz) specific for XDH, Nox2, Nox4 or a scrambled (control) sequence at a concentration of 100 pmol/ml using DharmaFECT 2 (Dharmacon Research) transfection reagent. Transfected cells were cultured in complete medium for 48 h before exposure to IH.

### Immunoblot Assay

Cell or tissue extracts (20 μg) were fractionated by polyacrylamide-SDS gel electrophoresis and immunoblotted with anti-HIF-2α (Novus Biologicals; NB100-122; 1/1000), (Acris Antibodies; AP23352PU-N; 1/1000), XDH (Santa Cruz; #sc-20991; 1/300), Nox2 (Santa Cruz, #sc-5827; 1/1000), Nox4 (Novus Biologicals, # NB110-58849; 1/1000), and tubulin (Sigma, # T 6199; 1/3000) antibodies as described [13].

### Measurements of XO Activity

Amplex Red Xanthine/Xanthine oxidase assay kit (Molecular Probes) was used to monitor to XO activity. Cell/tissue lysates were incubated with a reaction mixture containing hypoxanthine, Horse readish Peroxidase (HRP) and Amplex Red. H_2_O_2_ generated reacts with Amplex Red in the presence of HRP to generate red-fluorescent oxidation product resorufin. The flurosence was measured by excitation at 530 and emission at 590 nm. Concentration of XO in the samples is determined from a standard curve and expressed as XO mU/mg protein.

### Measurements of Calpain Activity

Calpain activity was measured using Innozyme™ calpain1/2 Kit (Calbiochem # CBA054) as per manufacturer’s instructions. Briefly, following IH exposure, cells were lysed in 50 mM Tris-Cl (pH 7.4), 0.5 mM EDTA, 0.05% β-mercaptoethanol, 1 mm PMSF and protease inhibitor cocktail (Sigma, MO) and calpain activity was measured using purified calpain as standard. Enzyme activity was normalized to protein content and expressed as percent of normoxic controls.

### Measurement of [Ca^2+^]_i_

[Ca^2+^]_i_ was monitored in PC12 cells using Fura-2 AM as described previously [21]. Background fluorescence was subtracted from signals. Image intensity at 340 nm was divided by 380-nm image intensity to obtain the ratiometric image. Ratios were converted to free [Ca^2+^]_i_ using calibration curves constructed *in vitro* by adding Fura-2 (50 µM, free acid) to solutions containing known concentrations of Ca^2+^ (0–2000 nM).

### Measurements of Aconitase Activity

Mitochondrial and cytosol fractions were isolated from cells or adrenal medullary extracts by differential centrifugation as described [22]. Aconitase activity was measured in both the fractions using aconitase assay kit (Cayman chemical company; # 705502) as described. Protein concentration was estimated using Bio-Rad protein assay kit. The enzyme activities were expressed as nanomoles per minute per milligram of protein.

### Real Time RT-PCR Assay

PC12 cells exposed to normoxia, or IH were used to generate total RNA followed by first-strand cDNA. Aliquots of cDNA were used in quantitative real-time reverse transcription polymerase chain reaction (rt RT-PCR) with rat-specific primers detecting either XDH α or β-actin using SYBR as a fluorogenic binding dye as described previously [18].

### Measurements of Blood Pressure (BP) and Plasma Norepinephrine (NE)

Arterial blood pressure was monitored non-invasively by the “tail-cuff” method in unsedated rats as described previously [23]. Blood pressures were recorded before and after IH exposure, each animal serving as its own control. Arterial blood samples were collected from urethane anesthetized rats (1.2 gm/Kg; IP) in heparinized vials (heparin, 30 IU per ml; n = 6). Plasma was separated and NE was extracted with cis-diol-specific affinity gel, acylated and quantitated by competitive ELISA kit (Labor- Diagnostika Nord Gmbh & Co.KG).

### Statistical Analysis

Data were expressed as mean ± S.E.M from 3–5 independent experiments each performed in triplicate. Statistical analysis was performed by analysis of variance (ANOVA) and *p* values <0.05 were considered significant.

## Results

### HIF-2α Degradation by IH Requires ROS

**Figure 1:**
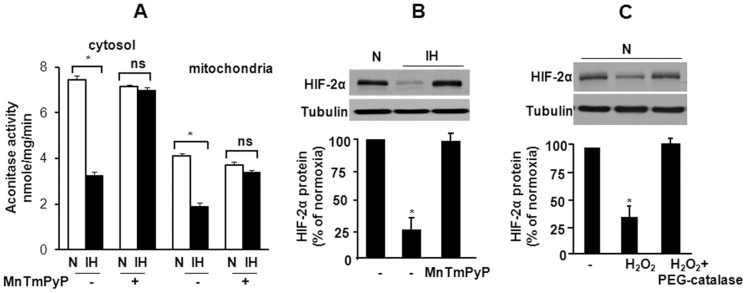
Reactive oxygen species (ROS) mediates IH-induced HIF-2α degradation. **A.** Acontiase activity levels were monitored as an index of ROS generation in cells exposed to normoxia (N) or IH with and without MnTmPyP (50 µM), an anti-oxidant. **B.** Representative immunoblot of HIF-2α in PC12 cells exposed to normoxia (N) or to 60 cycles of intermittent hypoxia (IH) with and without MnTmPyP. **C.** Effect of H_2_O_2_ on HIF-2α expression in PC12 cells exposed to normoxia with and without PEG-catalase (300 U/ml)**.** *p<0.05. n.s. not significant *p*>0.05.

HIF-1α activation by IH requires ROS signaling [13]. We therefore hypothesized that ROS also contribute to HIF-2α degradation by IH. PC12 cells were exposed to 60 cycles of IH consisting alternating cycles of hypoxia (1.5% O_2_ for 30 s) and re-oxygenation (20% O_2_ for 4 min). ROS generation was determined by monitoring the aconitase enzyme activity in cytosolic and mitochondrial fractions and HIF-2α protein levels were determined by western blots as described previously [18]. In IH exposed cells, aconitase activity decreased in cytosolic and mitochondrial fractions, and HIF-2α protein levels decreased (Fig. 1A–B). MnTmPyP, a membrane permeable ROS scavenger prevented IH-induced changes in the aconitase activity and HIF-2α degradation (Fig. 1A–B). Conversely, treating control PC12 cells with H_2_O_2_, an established ROS mimetic, decreased HIF-2α protein expression and PEG-catalase, which degrades H_2_O_2_ prevented this effect (Fig. 1C).

### NADPH Oxidases (Nox) are Not Required for IH-evoked HIF-2α Degradation

**Figure 2:**
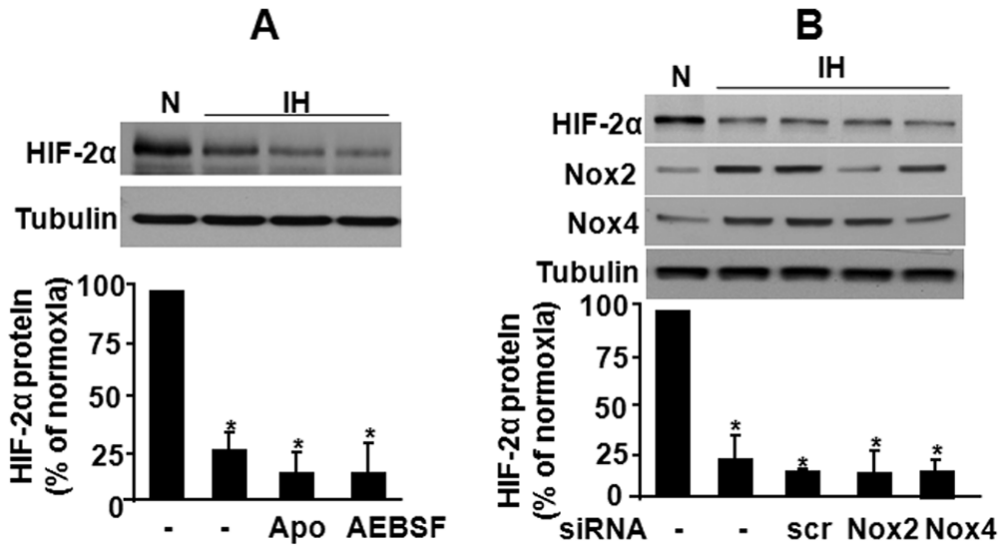
Role of NADPH oxidases in IH-induced HIF-2α degradation. **A.** Effect of NADPH oxidase (Nox) inhibitors Apocynin (Apo, 1 mM) and AEBSF (15 µM) on HIF-2α protein following exposure to IH. **B.** HIF-2α expression in PC12 cells transfected with Nox2 and Nox4 siRNA and exposed to normoxia (N) or IH. Tubulin expression was monitored as control for protein loading. Bottom panels of A and B represent average data of densitometric analysis of the immunoblots presented as mean ± S.E.M from three independent experiments. *p<0.05; n.s. not significant, *p*>0.05.

We then examined the source of ROS generation contributing to HIF-2α degradation by IH. NADPH oxidases (Nox), especially Nox2 and Nox4 are one of the major sources of IH-induced ROS generation in PC12 cells [24]. To assess the role of Nox-derived ROS, cells were exposed to IH in presence of either apocynin, or 4-(2-aminoethyl) benzenesulfonyl fluoride hydrochloride (AEBSF), two structurally distinct inhibitors of Nox isoforms. Neither apocynin nor AEBSF were able to prevent HIF-2α degradation by IH (Fig. 2A). To further assess the role of Nox isoforms, PC12 cells were transfected with a small interfering RNA (siRNA) targeted against either Nox2 (siNox2) or Nox4 (siNox4). IH-induced HIF-2α degradation was not inhibited in cells transfected with either siNox2 or siNox4 RNA (Fig. 2B).

### Xanthine Oxidase (XO) Mediates HIF-2α Degradation by IH

**Figure 3:**
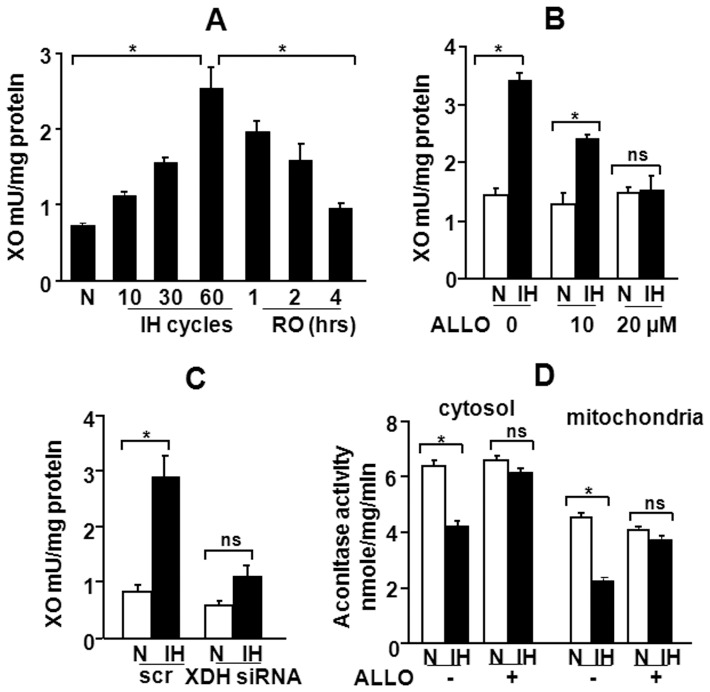
Xanthine oxidase activation by IH contributes to increased ROS levels. **A.** Effect of IH on XO activity. XO activity was determined in PC12 cells exposed to increasing cycles of intermittent hypoxia (IH_10,30,60_) and after re-oxygenation following exposure to IH_60_ **. B–C.** Effect of allopurinol (ALLO), a XO inhibitor and silencing of XO by siRNA on XO activity in cells exposed to IH. **D.** Acontiase activity levels were monitored as an index of ROS generation in cells exposed to normoxia (N) or IH with and without ALLO (20 µM) treatment. *p<0.05; n.s. not significant, *p*>0.05.

Xanthine oxidoreductase system is another major source of cellular ROS [25]–[27]. It is comprised of xanthine dehydrogenase (XDH) and xanthine oxidase (XO), and catalyze the oxidation of purines generating superoxide radicals as by product. XDH utilizes NAD^+^ as an electron acceptor, whereas molecular oxygen is a preferred electron acceptor for XO. Given that IH leads to changes in O_2_ levels, we hypothesized that ROS generated by XO contributes to HIF-2α degradation by IH. To test this possibility, we first determined the effect of IH on XO activity. XO activity increased in a stimulus-dependent manner as the duration of IH was increased from 10 to 30 to 60 cycles and returned to base line values within 4 hrs after terminating IH (Fig. 3A). IH-induced XO activity was prevented by allopurinol (ALLO), an inhibitor of XO (Fig. 3B) and was absent in cells transfected with siRNA targeted to xanthine dehydrogenease (XDH), the precursor of XO (Fig. 3C). IH-induced ROS generation was prevented in cells treated with ALLO (Fig. 3D).

**Figure 4:**
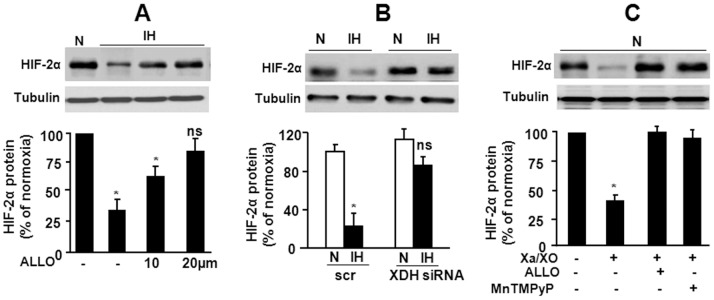
Xanthine Oxidase (XO) activity mediates HIF-2α degradation by IH. **A–B** Representative immunoblot of HIF-2α expression in cells exposed to IH in the presence of ALLO (A), and in cells transfected with XDH siRNA (B). **C** HIF-2α degradation in PC12cells treated with Xanthine (Xa)/XO (250 uM/0.01 U/ml) under normoxia, and the effect of MnTmPyP or ALLO co-treatment, respectively. *Bottom panels* of A, B and C show quantitative data of densitometric analysis presented as mean ± S.E.M. from 4 experiments, *p<0.05. n.s. not significant *p*>0.05.

To determine the contribution of XO to IH-induced HIF-2α degradation, cells were treated with ALLO and then were exposed to IH. ALLO prevented IH-evoked HIF-2α degradation in a dose-dependent manner (Fig. 4A). HIF-2α degradation by IH was absent in cells transfected with siRNA targeted to XDH (Fig. 4B). Conversely, activating XO with Xa/XO (250 µM/0.01 U/ml) markedly decreased HIF-2α protein in control PC12 cells, mimicking the effects of IH, and this effect was prevented by either ALLO, an inhibitor XO or by MnTmPyP, a ROS scavenger (Fig. 4C).

### Mechanisms Mediating XO Activation by IH

**Figure 5:**
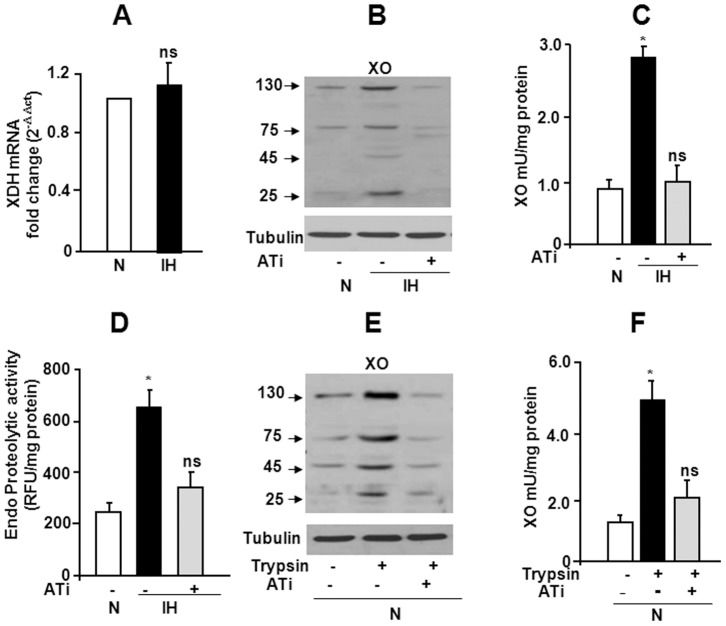
Mechanisms involved in IH-augmented XO activity. **A.** Xanthine dehydrogeanse (XDH) mRNA levels in PC12 cells exposed to normoxia (N) or IH. **B.** Representative immunoblot showing effect of trypsin protease inhibitor (ATi; 0.1 mg/ml) on IH-evoked XO protein expression. Tubulin expression was loaded as control for protein loading. **C.** XO activity in PC12 cells treated with trypsin inhibitor (ATi) and exposed to IH. **D.** Endoproteolytic activity expressed as relative fluoresence units (RFU)/mg protein in control and IH exposed cells with and ATi treatment. **E & F.** Effect of trypsin (0.1 ng/ml) on XO protein (E) and activity (F) with and without ATi treatment under normoxic conditions. *p<0.05. n.s. not significant. *p*>0.05.

The above results suggest that ROS generated by XO is critical for HIF-2α degradation by IH. We then investigated the mechanisms by which IH activates XO. To determine whether IH leads to transcriptional activation of XDH, the precursor of XO, quantitative real–time PCR (rt RT-PCR) analysis was performed on control and IH exposed cells. No significant change was observed in the XDH mRNA levels in IH exposed cells as compared to control cells (*P*>0.05; Fig. 5A).

Trypsin-like proteases mediate proteolytic conversion of XDH to XO [26]. Analysis of XO protein by western blot assay showed increased expression of three proteolytically processed products of XDH (25, 45 and 75 kDa) in lysates from IH exposed cells (Fig. 5B). Trypsin inhibitor (ATi) prevented the expression of XDH proteolytic products as well as XO activation by IH (Fig. 5B–C). Measurements of trypsin-like endoprotease activity revealed a 3-fold increase in IH exposed cells as compared to control cells and this effect was prevented by trypsin inhibitor ATi (Fig. 5D). Treating control PC 12 cells with trypsin increased the expression of XDH proteolytic products with concomitant increase in XO activity, and these effects were prevented by ATi (Fig. 5E–F).

### XO Mediates Calpain Activation by IH

**Figure 6:**
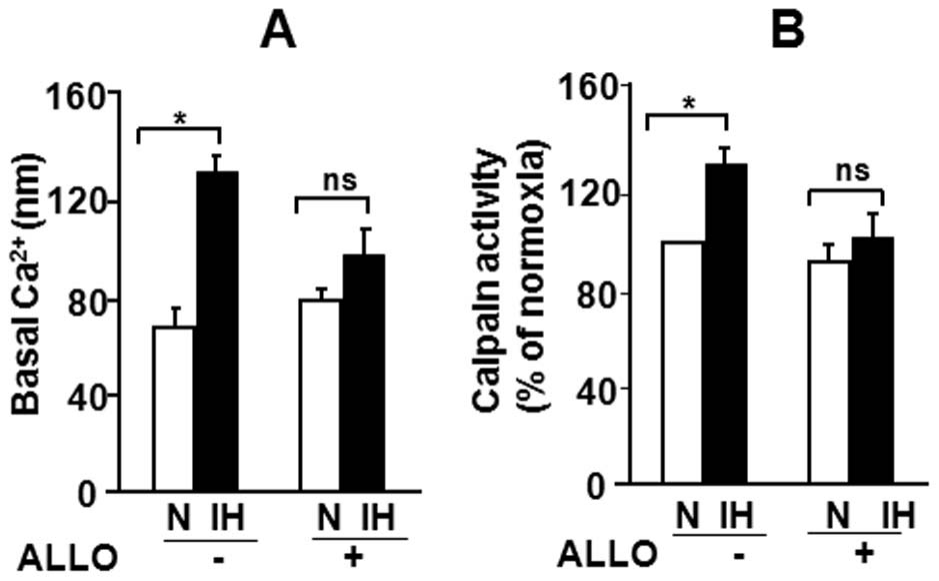
IH-induced changes in [Ca^2+^]_i_ and calpain activity are mediated by XO. **A.** Effect of ALLO treatment on IH augmented basal [Ca^2+^]_i_ levels in PC12 cells. **B.** Treatment of cells with ALLO prevents calpains activation by IH in PC12 cells. Data are presented as mean ± S.E.M from 3 independent experiments. *p<0.05; n.s. not significant. *p*>0.05.

We previously reported activation of calpains, which are Ca^2+^- dependent proteases mediate HIF-2α degradation by IH [18]. We therefore examined whether XO contributes to IH-evoked calpain activation. The effect of IH on [Ca^2+^]_i_ levels, a requisite for calpain activation along with calpain enzyme activity were determined in control and IH exposed cells in the presence of vehicle or ALLO. IH exposed cells exhibited elevated levels of basal [Ca^2+^]_i_ and increased calpain activity and these effects were absent in the presence of ALLO (Fig. 6 A–B).

### C-terminus is Required for HIF-2α Degradation by IH

**Figure 7:**
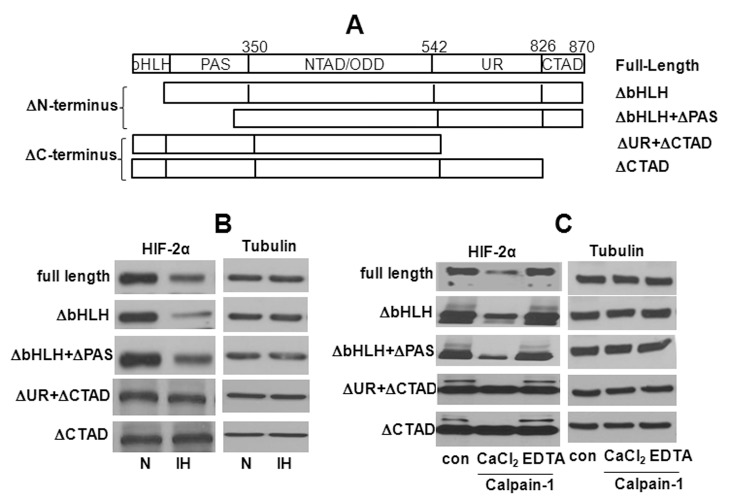
Involvement of N- terminus and C- terminus HIF-2α in IH-induced HIF-2α degradation by calpains. **A.** Schematic diagram showing the full length, N-terminus (ΔbHLH and ΔbHLH+ΔPAS) and C-terminus (ΔUR, ΔUR +ΔCTAD) deleted constructs. **B.** Western blot showing HIF-2α protein in PC12 cells transiently transfected with the HIF-2α full length and the two N- and C-terminus truncated constructs and exposed to normoxia (N) or IH. The N-terminus and C-terminus deleted HIF-2α proteins were detected with antibody raised against HIF-2α N-terminus (Acris Antibodies; AP23352PU-N) and C-terminus (Novus Biologicals; NB100-122) respectively. **C.** PC12 cell lysates expressing the N- and C-terminus deleted protein were incubated for 15 min with purified calpain-1 (3 mg/ml) in presence of 1 mM CaCl_2_ or 1 mM CaCl_2_+2 mM EDTA and HIF-2α protein was analyzed by western blot.

The N-terminus of HIF-2α protein is composed of basic helix-loop-helix (bHLH) and PER-ARNT-SIM (PAS) domain, and the C-terminus comprises of a unique region (UR) of highly divergent sequence and C-terminal transactivation domain (CTAD). Calpain cleavage prediction using multiple Kernel learning [28] from Calpain Modulatory Proteolysis Data Base (CaMPDB) identified three potential calpain binding sites in HIF-2α protein, one at the amino-terminus (N-terminus) and the other two at the carboxy-terminus (C-terminus). The contribution of N-terminus and C-terminus to IH-induced HIF-2α degradation by calpains was determined. PC12 cells transfected with full length HIF-2α plasmid or plasmids with bHLH or bHLH +PAS domain deletions at the N- terminus or UR +CTAD or CTD deletions at the C- terminus (Fig. 7A). HIF-2α degradation by IH was seen in cells expressing full length HIF-2α, and ΔbHLH or ΔbHLH +PAS at the N-terminus (Fig. 7B). In striking contrast, HIF-2α degradation by IH was absent in cells transfected with plasmids with C-terminus deletion of UR +CTAD or CTD (Fig. 7B), suggesting that CTAD contributes to HIF-2α degradation by IH.

To further establish that CTAD of HIF-2α protein is a substrate for calpain 1, cell lysates from normoxic PC12 cells transfected with UR +CTAD or CTD deleted plasmids of the C-terminus of HIF-2α were incubated with purified calpain 1 (3 mg/ml) in presence of CaCl_2_ (1 mM) with and without the Ca^2+^ chelator EDTA (2 mM). Control experiments were performed on cells transfected with either full length or bHLH or bHLH +PAS deleted N- terminus plasmids. HIF-2α was completely degraded when cell lysates expressing full length HIF-2α were incubated as brief as 15 min with calpain 1 and CaCl_2_. Addition of EDTA prevented this effect (Fig. 7C **)**. Similar proteolytic degradation of HIF-2α was also observed with the two N-terminus deleted HIF-2α constructs. However, cell lysates from C-terminus deleted HIF-2α plasmids were resistant to degradation by exogenous calpain (Fig. 7C).

### Role of XO Activity in IH-induced Autonomic Dysfunction

**Figure 8:**
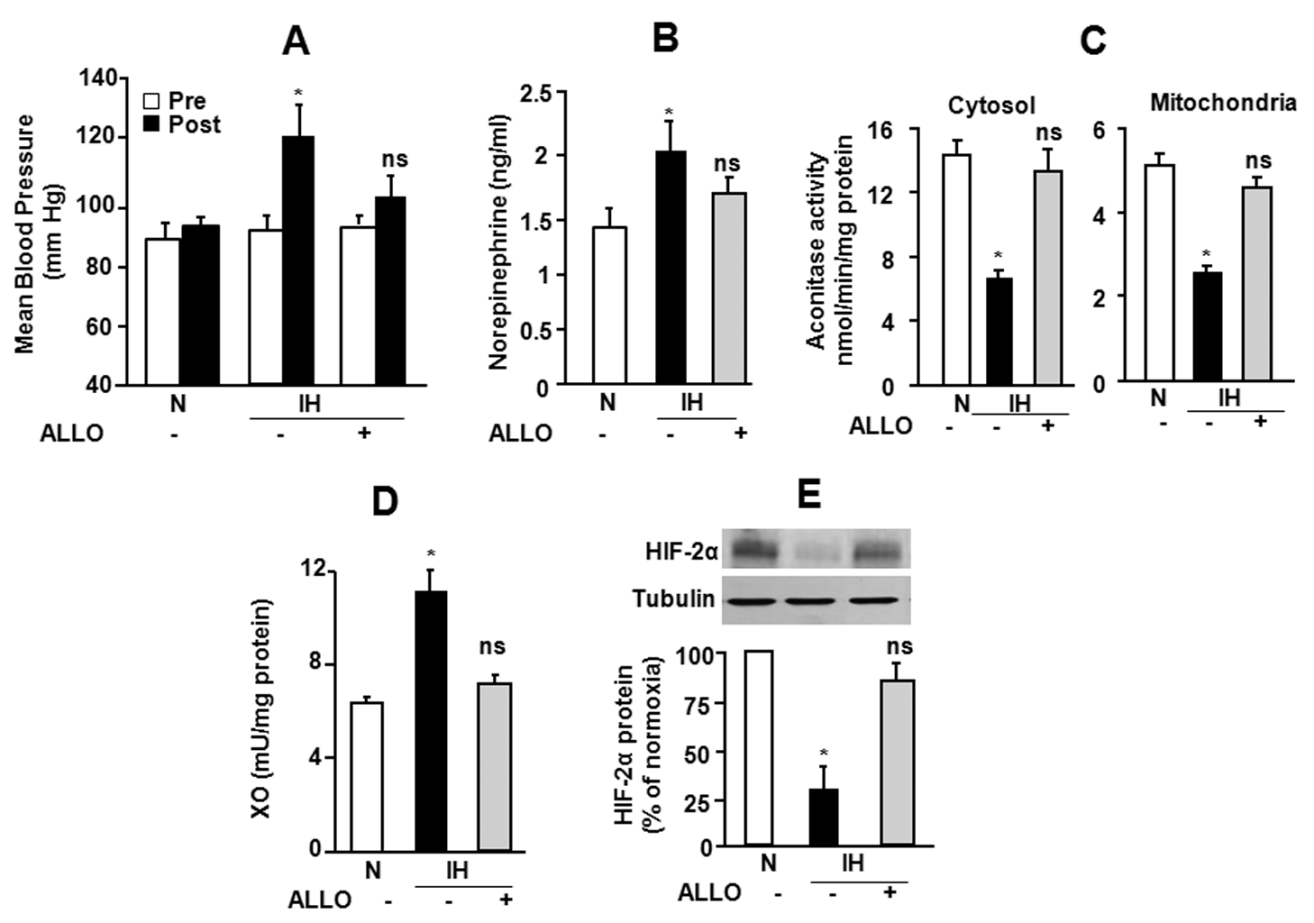
Effect of XO inhibitor allopurinol (ALLO) on IH-induced autonomic dysfunction. Adult rats were exposed to 10 days of IH and were treated daily with either vehicle (IH) or ALLO (65 mg/Kg/day; oral gavage; IH+ALLO). Control experiments were performed on rats exposed to normoxia (N). **A–B** Increases in mean arterial blood pressure and plasma norepinephrine levels. **C**. Decreased aconitase activity (index of ROS generation) levels, **D** Increased XO activity and **E.** HIF-2α degradation in adrenal medullae. Data presented in A–E are mean ± S.E.M. from 8 rats in each group. *p<0.05. n.s. not significant p>0.05.

HIF-2α degradation increases oxidative stress, which contribute to autonomic dysfunction in rodents exposed to IH [18]. We examined whether blocking HIF-2α degradation by XO inhibitor restore autonomic function in IH exposed rats. Adult rats were treated with daily oral gavage of either ALLO (65 mg/Kg/day) or vehicle for 10 days prior to exposing them to daily regimen of 8 h of IH. Blood pressures were monitored in conscious rats before (pre) and after 10 days of CIH (post). Vehicle treated IH rats exhibited significantly increased mean blood pressures (Fig. 8A), which was due to significant elevations in systolic and diastolic blood pressures (P<0.01), and was associated with elevated plasma norepinephrine levels, an index of sympathetic activation (Fig. 8B). Adrenal medulla is a major source of catecholamines, and adrenal medullectomy prevents IH-induced hypertension [29]. Therefore, cytosolic and mitochondrial aconitase activity (index of ROS levels), XO activity and HIF-2α protein expressions were determined in adrenal medullae from vehicle and ALLO treated rats exposed to IH. Adrenal medulla from vehicle treated rats exposed to IH exhibited elevated ROS levels as evidenced by decreased cytosolic and mitochondrial aconitase activity, with concomitant increase in XO activity and decreased HIF-2α protein expression (Fig. 8C–E). ALLO treatment restored blood pressures, normalized plasma norepinephrine levels prevented the increased ROS levels as well as XO activity and HIF-2α degradation in adrenal medullae from IH exposed rats (Fig. 8A–E).

## Discussion

Present study delineated the signaling mechanisms mediating HIF-2α protein degradation by IH. Consistent with previous reports [18], we found decreased HIF-2α protein expression in cell cultures and in adrenal medulla from rats exposed to IH. Our results further demonstrate that HIF-2α degradation by IH was prevented by ROS scavengers and a ROS mimetic decreased HIF-2α expression in control cells exposed to normoxia. These findings demonstrate that ROS signaling is critical for mediating the effects IH on HIF-2α protein. Although IH activates Nox2 and Nox4 in cell cultures and in rodents [13], neither pharmacological blockade of Nox isoforms nor selective genetic knock down of Nox2 or Nox4 by siRNA approach were able to block HIF-2α degradation by IH. These observations suggest that ROS generated by sources other than Nox2 or Nox4 contribute to HIF-2α degradation by IH.

The following findings demonstrate that activation of XO and the resulting ROS mediate HIF-2α degradation by IH. First, IH activated XO and pharmacological blockade of XO prevented ROS generation by IH. Second, genetic silencing of XO or blockade of XO by ALLO prevented IH-induced HIF-2α degradation. Third, activation of XO decreased HIF-2α expression in control cells, mimicking the effects of IH. Our results further provide insights into the mechanism of XO activation by IH. mRNA expression of XDH, the precursor of XO was unaltered by IH, suggesting that transcriptional regulation does not account for XO activation. The following findings suggest that proteolytic processing of XDH contributes to XO activation. First, IH exposure resulted in increased proteolytic products of XO. Second, trypsin inhibitor blocked the generation of proteolytic products and XO activation by IH. Third, treatment of PC12 cells under normoxic conditions with trypsin mimicked the effects of IH. Fourth, IH increased trypsin-like endoprotease activity, which was blocked by trypsin inhibitor. These findings are reminiscent of an early study showing that XDH is converted to XO within minutes in response to ischemia, which is blocked by inhibiting proteolysis with a trypsin inhibitor [30], [31]. In addition to proteolytic processing, IH might also activate XO via sulphydryl oxidation, a possibility that requires further study.

How might XO contribute to HIF-2α degradation by IH? Based on our previous study [18], we hypothesized that ROS generated by XO increases [Ca^2+^]_i_ levels leading to calpain activation resulting in HIF-2α degradation. Consistent with this possibility, we found that inhibition of XO prevented IH-induced calpain activity and this effect was associated with the absence of increase in [Ca^2+^]_i_ levels. Calpain Modulatory Proteolysis Data Base identified three potential calpain binding sites in HIF-2α protein, one at the amino-terminus (N-terminus) and the other two at the carboxy-terminus (C-terminus). The C-terminus transctivation domain (CTAD) controls the transcriptional activity of HIF-2α by recruiting histone acetyl transferases p300 and CBP which act as transcriptional activators. Our data with forced expression of plasmids with N-terminus and C-terminus deletions demonstrated that CTAD is the target for calpain-mediated HIF-2α degradation.

**Figure 9:**
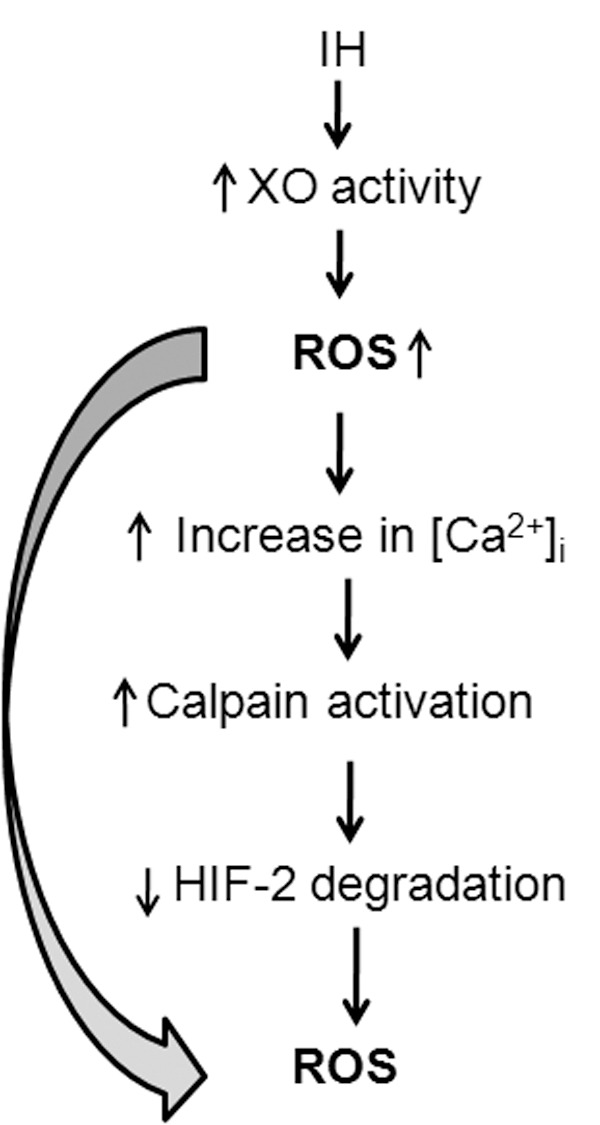
Schematic representation of IH effects on XO activity which mediates IH-induced HIF-2α degradation leading to positive feed forward ROS-induced ROS mechanism.

It is interesting to note that HIF-2α regulates genes encoding anti-oxidant enzymes, notably SOD-2 and preventing HIF-2α degradation blocks IH-induced oxidative stress. Since, ROS generated by XO are required for IH-induced HIF-2α degradation, it is likely that transient ROS generation by XO triggers a more persistent ROS production by HIF-2-dependent insufficient transcriptional activation of anti-oxidant enzyme genes i.e., ROS-induced ROS mechanism (Fig. 9). This novel positive feed-forward mechanism identified in this study might explain how IH induces long-lasting oxidative stress.

Our results further demonstrate that XO initiated ROS-dependent degradation of HIF-2α occurs not only in cell cultures but also in tissues (adrenal medulla) of IH exposed rodents. IH activated XO, as well as elevated ROS levels in adrenal medulla with concomitant decrease in HIF-2α, and all these effects were effectively blocked by ALLO, a XO inhibitor. Previous studies showed that IH-induced oxidative stress contributes to autonomic dysfunction including hypertension in rodents [18]. Remarkably, ALLO prevented IH-induced hypertension and elevated plasma norepinephrine levels in rats. These finding suggest that activation of XO triggers a sequel leading to autonomic dysfunction associated with IH. Indeed, XO has been implicated in mediating pathologies associated with pulmonary [32] and salt-induced hypertension [33] as well as in ischemia/re-perfusion injury [34].

In addition to cardiovascular abnormalities, IH associated with OSA leads to inflammation [35]. Recent studies have shown that HIF-1 contribute to continuous hypoxia-induced inflammation [36], [37]. Down regulation of HIF-2α increases HIF-1α expression [16]. It is likely that XO-dependent down regulation of HIF-2α either directly or indirectly via HIF-1α might contribute to IH-induced inflammatory responses, a possibility that remains to be investigated. Besides HIF’s, IH is also known to regulate other transcription factors like NF-kB [38] and possibly Nrf2 [39], [40]. Whether ROS plays a role in their activation or their contribution to IH-induced oxidative stress, need to be examined.
